# Nursing Home Palliative Care During the Pandemic: Directions for the Future

**DOI:** 10.1093/geroni/igac030

**Published:** 2022-05-09

**Authors:** Kacy Ninteau, Christine E Bishop

**Affiliations:** Department of Psychosocial Oncology and Palliative Care, Dana-Farber Cancer Institute, Boston, Massachusetts, USA; Heller School for Social Policy and Management, Brandeis University, Waltham, Massachusetts, USA

**Keywords:** Advance directives, Nursing staff, Serious illness, Symptom management

## Abstract

**Background and Objectives:**

Palliative care addresses physical, emotional, psychological, and spiritual suffering that accompanies serious illness. Emphasis on symptom management and goals of care is especially valuable for seriously ill nursing home residents. We investigated barriers to nursing home palliative care provision highlighted by the coronavirus disease 2019 (COVID-19) pandemic and the solutions nursing home staff used to provide care in the face of those barriers.

**Research Design and Methods:**

For this descriptive qualitative study, seven Massachusetts nursing home directors of nursing were interviewed remotely about palliative care provision before and during the COVID-19 pandemic. Interview data were analyzed using thematic analysis.

**Results:**

Before the pandemic, palliative care was delivered primarily by nursing home staff depending on formal and informal consultations from palliative care specialists affiliated with hospice providers. When COVID-19 lockdowns precluded these consultations, nursing staff did their best to provide palliative care, but were often overwhelmed by shortfalls in resources, resident decline brought on by isolation and COVID-19 itself, and a sense that their expertise was lacking. Advance care planning conversations focused on hospitalization decisions and options for care given resource constraints. Nevertheless, nursing staff discovered previously untapped capacity to provide palliative care on-site as part of standard care, building trust of residents and families.

**Discussion and Implications:**

Nursing staff rose to the palliative care challenge during the COVID-19 pandemic, albeit with great effort. Consistent with prepandemic analysis, we conclude that nursing home payment and quality standards should support development of in-house staff capacity to deliver palliative care while expanding access to the formal consultations and family involvement that were restricted by the pandemic. Future research should be directed to evaluating initiatives that pursue these aims.

**Translational Significance:** Nursing homes often fall short in their capacity to provide palliative care, whether relying on internal or external resources. The multiple pathways for palliative care before and during the coronavirus disease 2019 pandemic suggest ways to enhance the provision of high-quality palliative care in nursing homes. Supporting in-house capacity to alleviate symptoms and crystallize evolving goals of care while expanding access to external consultation would foster an integrated approach to resident-centered goals of care.

## Background and Objectives

Palliative care is an approach to serious illness care that aims to improve patient quality of life by identifying and treating disease symptoms, pain, and emotional or psychological distress. In its focus on patient wishes and goals of care and its individualized response to distressing symptoms, palliative care represents the epitome of person-centered clinical care in the nursing home and has been identified as “one and the same” with good quality nursing home care (Ersek et al., 2022). The strictures that the coronavirus disease 2019 (COVID-19) pandemic imposed on U.S. nursing homes threw into sharp relief both barriers to and opportunities for better integration of palliative care into nursing home practice to achieve more person-centered care.

Nursing home residents are often diagnosed with multiple chronic conditions and receive both treatment and symptom relief from medical and nursing care. Residents who receive palliative care have been found to have better quality of life, experience fewer hospitalizations, and are less likely to die while hospitalized (Ersek et al., 2022; Harrison et al., 2021; Martin et al., 2016; Miller, Dahal, et al., 2016; Miller et al., 2017; Temel et al., 2010). Some nursing homes have the internal capacity to deliver this care, so that staff with trusted relationships elicit goals of care and provide symptom relief (Carlson et al., 2011; Carpenter et al., 2020). But many nursing homes rely on outside hospice and palliative providers (Carlson et al., 2011; Meier, 2015).

Although hospice services, which are a main avenue for palliative care delivery in nursing homes, often provide essential support and end-of-life care, use of the Medicare Hospice Benefit is restricted to residents who have a prognosis of 6 months or less and are willing to forgo any treatment with a curative intent (Centers for Medicare and Medicaid Services, 2021). In some areas, nursing home residents may be able to access palliative care through consultations with external specialists. These visits may include goals-of-care conversations, psychosocial support, symptom management, or referral to hospice services. Access to specialists is limited by clinician shortages and limited contracts between nursing homes and outside provider groups (Carlson et al., 2011; Carpenter et al., 2020; Ersek et al., 2022).

The novel coronavirus caused distressing, painful symptoms and elevated risk of death for nursing home residents while simultaneously isolating nursing homes and their residents from usual sources of external support and vital social contact. The prevalence of comorbidities, including dementia, cardiovascular disease, chronic respiratory disease, and frailty, increased the likelihood that residents would experience severe cases of COVID-19 or die (Su et al., 2021; Thompson et al., 2020). Congregate living in itself made it difficult to shield residents once even one resident or staff member was infected (Ouslander & Grabowski, 2020; Su et al., 2021). Reports from the front lines document decline in residents’ overall well-being, increase in depressive symptoms, weight loss, loneliness, and clinician burnout due to high stress and overwhelming workloads (Levere et al., 2021; White et al., 2021).

The goal of our study was to learn from the COVID-19 crisis. We sought to investigate how nursing home staff navigated palliative care delivery in the face of nursing home lockdowns, restricted access to external providers, limited resources, and the COVID-19 disease itself. Our aim was to uncover the barriers to palliative care at work during the COVID-19 pandemic, as well as to highlight novel approaches that in-house staff took to sustain care for residents. In this article, we report the observations of nursing home directors of nursing (DONs) whose residents needed palliative care during COVID-19. We build on their insights to propose directions for future research, policy, and practice.

## Research Design and Methods

**Figure 1.**
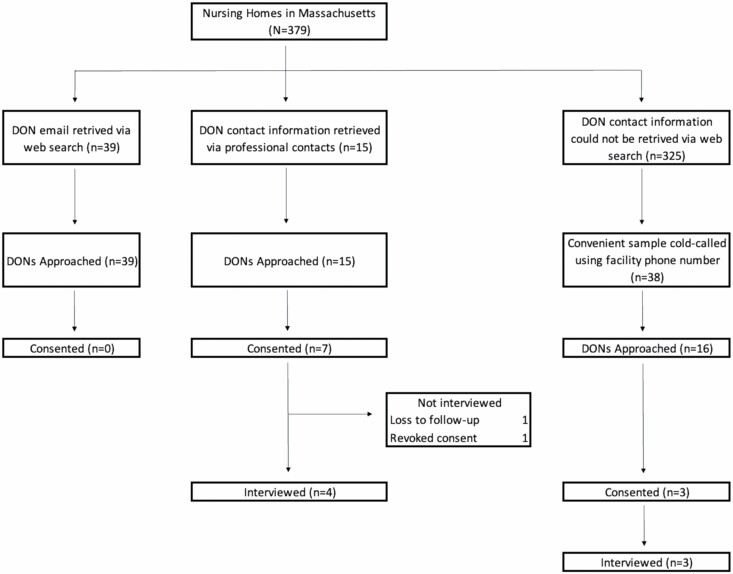
Recruitment Schematic. DON = director of nursing.

For this small, descriptive study, semistructured interviews of nursing home DONs were analyzed using thematic analysis to capture how nursing homes addressed palliative care needs during the pandemic. The study group was comprised of DONs working in Massachusetts nursing homes for at least 6 months. The study design was finalized in Fall 2020, as pandemic case numbers were falling and vaccines were coming on line. Participants were recruited between January 2021 and July 2021 during an unexpected upsurge in COVID-19 cases. Even with a goal of interviewing only 10 subjects, it proved difficult to recruit interview subjects during the second wave of the pandemic in Massachusetts, a problem not anticipated in the study design (Figure 1).

A list of the 379 nursing homes in the state was extracted from the Massachusetts Department of Public Health. We approached 54 DONs whose email addresses were published on their facilities’ websites, and when this approach did not yield any participants, used emails located through professional contacts. Using telephone numbers from the state list, we cold-called another group of facilities to locate DONs’ contact information in an attempt to add nonprofit homes, which were underrepresented in the first group to respond. Of the 92 DONs contacted, nine agreed to participate. One DON revoked consent before the interview, citing an overwhelming schedule, and another DON did not attend the interview session and was considered lost to follow-up, for a total of seven subjects.

The DONs’ nursing homes varied by ownership, size, and Medicaid utilization (Table 1) and were located in 5 of the 14 counties in Massachusetts, in the central and western parts of the state as well as in the greater Boston area. This study was approved by the Brandeis University Institutional Review Board. Participants were sent an informed consent form via email, which they signed before the interview began. They were reminded at the start of the interview that participation in the study was voluntary and that they could skip any question they did not wish to answer.

**Table 1.** Characteristics of Nursing Homes Employing Study Directors of Nursing

DONs	Nursing home profit status	Nursing home size^a^	% Medicaid^b^
DON 1	For profit	Medium	Medium
DON 2	For profit	Large	Medium
DON 3	For profit	Medium	High
DON 4	For profit	Medium	High
DON 5	For profit	Large	Medium
DON 6	Nonprofit	Large	Medium
DON 7	Nonprofit	Large	Medium

*Notes*: DON = director of nursing. All nursing homes belonged to multifacility corporations, were certified for both Medicare and Medicaid services, and are located in 5 of the 14 Massachusetts counties.

^a^Size: medium = 70–119 beds; large = 120+ beds.

^b^% Medicaid: proportion of facility residents whose primary financial support is Medicaid. Medium = 50%–79%; high = 80%–100%.

*Source:* LTCFocus (2021).

Between February 2021 and July 2021, K.N. conducted semistructured interviews with seven participants. Zoom video-conferencing technology was used to respect the need for social distancing during the COVID-19 pandemic. Audio of the interviews was recorded on a personal recording device. Each interview lasted 20–30 min and followed an interview guide (Supplementary Materials). The interview protocol had three sections: palliative care delivery before the COVID-19 pandemic, advance care planning, and palliative care during the COVID-19 pandemic.

K.N. analyzed the data using a thematic analysis approach. This approach best fit the narrative nature of the interview data and allowed for both inductive and deductive interpretation (Supplemental Materials; Schoenberg & McAuley, 2007; Vaismoradi et al., 2013). The interviews were transcribed verbatim. The transcripts were read at multiple time points to achieve immersion (Vaismoradi et al., 2013). After careful reading, noteworthy phrases were highlighted and detailed notes on first impressions were made to capture meaningful, recurrent ideas, and key issues (Vaismoradi et al., 2013). The data were coded using conceptual codes, which were then classified, compared, and organized into subthemes (Supplementary Materials; Vaismoradi et al., 2016). Codes and illustrative quotes within each subtheme were used to abstract themes. The themes were constructed to highlight the changes in palliative care delivery that occurred during the COVID-19 pandemic, as well as to organize the narratives around the dimensions of palliative care delivery.

## Results

From the interview data, we abstracted five themes that highlight key aspects of palliative care delivery and how these changed in response to the COVID-19 pandemic. Table 2 outlines these themes and presents subthemes and illustrative quotes for each.

**Table 2.** Illustrative Quotes From DONs Stratified by Theme and Subtheme

Theme and subtheme	Quotes
Prepandemic palliative care practices	
How DONs define palliative care	“I see palliative care as, ‘can you help me make them more comfortable?’ Their life isn’t ending tomorrow ... if I can just get by on palliative consults to make them comfortable and let them continue their daily living, that’s great.” “I think that palliative care is a nicer way of putting it than hospice, I think there’s still a stigma associated with hospice that rest of the world doesn’t seem to understand, which is why it’s a tougher sale of some families.” “Palliative care is more or less the facility running the plan of care for the patient.” “If they are declining, we focus on what they want for their goal. If they want to continue going out to the doctors or follow-up treatment or radiation, that’s what we do. We support them in what they choose to do, to have. Everyone’s palliative care is different” “We could actually say to family, we think we should really look at the palliative route right now. We can do lot here if they do have their 85th UTI, they don’t need to go into the hospital, we can treat in place ... and I think families are a little relieved when we say that we can do things here because they still want to be aggressive but yet not to the point that they are in ICU care and [there is] disruption from normal routine.”
Pain management	“Palliative care coming in and hospice coming in, they pretty much have their own standards, usually they go for the big guns ... like most everybody gets morphine coming in for hospice.” “There are possibilities that pain might not be managed very well and they’re heavily relying on narcotics. Well, we could use alternative pain measures that could work and be just as effect for say nerve pain or different types of pain that doesn’t necessarily need a narcotic.”
Role of outside palliative care and hospice providers	“if [pain] could not be breached, I would talk to some palliative nurses that I know with hospice to get some treatment modalities or try to figure out what is a good combo. I’m not as skilled as they are.” “[Nurse practitioners from hospice group] could come do palliative care for me. They were in the building so they would screen somebody else for me.” “I have switched hospice groups because I find them to be too cautious and timid.” “If we had a tricky case that [the resident] didn’t want a hospice agency in on and were just looking for some ideas or brainstorming, they [hospice providers] would certainly collaborate with us just off the record.”
Staff education	“I don’t think nursing is really trained to look at pain as the 5th vital sign or whatever. I just don’t think they do it well.” “I wish I was more skilled. I do think [palliative care] is something that should probably be looked at when they’re teaching in schools.” “[Contact with outside palliative care providers] is good education for my new nurses, as well, to always keep a handle on how to assess pain and adequately treat pain.” “I had certain nurses that would [learn from physicians and nurse practitioners] and then would make their own suggestions, then we would talk about it. I had other nurses that would say to me, ‘well they didn’t have any pain.’ ‘Well how come she’s squirming?’”
Palliative care delivery during COVID-19 pandemic	
Role designation with loss of access to outside palliative care	“When we had hospice people coming in ... they also bought into the team of the center so they would have a patient in bed A and they would also visit with bed B and that’s been restricted since COVID.” “[Nurse practitioners with DON] have been trying some not narcotics but just some simple things that might make a difference. Like I said, the heating pad and rehab ... a couple different modalities.” “Our medical director is hospice and palliative care certified, so we do have that back-up. We are lucky to have him, so honestly, palliative care, getting someone in here for palliative care [COVID] is very low on my list.” “I think that our medical director is definitely comfortable in end-of-life care and meeting those needs, but also our nurse practitioner, he is here every day, and he has those conversations, he involves the floor staff with it, I really think he does on the spot education.” “The nursing [staff] and I are doing their comfort care ... if I need guidance [from palliative care or hospice specialists], you do have to contract with them, but all of the contracts have gone to the wayside. So, the physicians and nurses come together for pain management or anything else we can suggest to help somebody out and be there for them.”
Care for residents with COVID-19	“Honestly it seemed to affect more of the healthy ones. It did affect a couple of the palliative patients, a portion of it was that, but with the portion of younger ones that really kind of took me back.” “As a facility, we missed the ones that didn’t have a respiratory component because we learned now there was no respiratory [signs], it was just a migraine or diarrhea and things like that.” “I think we learned as we went along. The people who got in trouble were the ones who got into a dehydrated state because for some reason COVID just takes away your appetite ... they didn’t have a lot of that ‘I can’t breathe’ stuff,’ it was a lot of the GI stuff that we missed.” “Care was really just making sure people were comfortable. After all attempts to turn them around or get them out of this situation, if they were not improving based upon what we did, then the conversation would shift to comfort, whether that means getting them something to ease their suffering or pain or just to calm them a little bit.”
Resource availability	“We’re able to treat better in-house I feel than we were at the beginning of the pandemic. We have the respiratory therapist available. Our pharmacy has made more meds available to use. Where before there was such a shortage on everything because everybody needed it at once, but I think we’ve kind of caught up and that helps.” “Without N95, it was difficult to use nebulizers and we switched to inhalers.”
Eliciting resident goals of care during the pandemic	
Staff communication with residents and families	“The doctors have been communicating well with [families] about prognosis and I think the families appreciate that.” “If I’ve had a difficult time with the patient [around care planning], well I mean with the family member, I’ve had the physician call and have a discussion with them to see if he can make them understand, just so that they are informed.” “In the height of COVID, we really took a deep dive into every single resident’s advance directive and some of them were changed because of that. We mostly changed the transfer to hospital status or intubation status.”
Care planning for COVID-19-positive resident	“We are more apt to have those palliative care kinds of conversations because we really don’t know how COVID affects everybody differently. Some people are completely asymptomatic, and some people are maybe a little symptomatic today have a little cough and the next day their pulse ox is in the 60s”
Hospitalization	“They can be very aggressive in the hospitals we are working with the hospitals now to try and have them honor what the palliative plan is or if we’re having a conversation with the family members and they’re not quite there to make palliative, we ask the hospitals to step in.” “We would say that they are the lowest of low for the totem pole to even get seen. You could be on a stretcher for hours in the emergency room is this something that you want to actually do?” “A couple of patients went out just to the ER, based upon their medical diagnosis, made them palliative, they didn’t treat them, and they died alone. That part was hard.” “You learn so much going through it that at that point, I made the determination at the facility level that we were not sending anybody else out—it’s do or die at the facility level.”
Building trust between residents, families, and nursing home staff	“When the facility kind of does our own palliative care, obviously you can develop a relationship with the families and resident because you are the primary caregiver for them. It’s not bringing in somebody at the end. Sometimes it’s just a comfort level that the family would prefer to have the people they know and trust taking care of them versus having someone come in who doesn’t know them yet.” “We are bringing [families] into the discussion. We’ve said, ‘this was a good week’ or ‘this was a bad week’ or we’ve started to say, ‘there’s been changes since you last saw them, and this is what we suggest’ ... there’s been a couple that will say ‘no, not yet’ but if we say it’s time to try this or that ... they will say ‘well you know her better.’”
Impact of resident isolation on palliative care	“The most gut-wrenching thing was that family members couldn’t come in. And, you know, we love them up and stuff but I’m sure we’re not the same as their children and grandchildren.” “The residents don’t even recognize who you are under all of the equipment.” “Overall declines in health despite people recovering from COVID almost six months ago. I think that it is almost like a failure to thrive. It’s hard to have to have something to look forward to when you’re staring at the same four walls.” “We’ve definitely had more hospice than I’ve seen in my whole career lately and like I said its definitely that failure to thrive aspect.” “It’s very difficult to distinguish between somebody getting to a palliative point now because of COVID because of such isolation that they are subjected to.” “If someone is actively dying, we do allow families to come in. They do have to be screened and escorted down. We have to educate [them on] the infection control practices, provide them with the appropriate equipment ... it depends on what stage in the dying process that somebody is. If [death] is imminent, then we really allow them just as much time as they need.”

*Notes*: COVID-19 = coronavirus disease 2019; DON = director of nursing; ER = emergency room; GI = gastrointestinal; ICU = intensive care unit; UTI = urinary tract infection.

### Prepandemic Palliative Care Practices

DONs’ definitions of palliative care conform to standard definitions—care that manages symptoms to make a patient with serious illness more comfortable. Some of the DONs noted that palliative care consultations are used not only when a patient is near death but also when they need additional support to continue daily living. They distinguished a palliative care “track” from formal Medicare-supported hospice enrollment, which requires terminally ill residents to forgo disease-directed treatment. Many residents experiencing severe pain and symptoms of serious illness request the palliative care track rather than hospice to continue to receive aggressive treatment, to retain the potential to be hospitalized, or to avoid adding more providers to their care team when they are comfortable with the care they are receiving from in-house staff.

Other DONs said that palliative care and Medicare-supported hospice care differ only in that palliative care does not involve an external hospice provider. When some residents and their families have trouble acknowledging futility of treatment, an in-house nurse practitioner or medical director may evaluate the resident for palliative care, because hospice is not authorized.

Advance care planning conversations are used to determine whether it is appropriate for a patient to be on a palliative care track. Ideally, the resident, family, and the resident’s medical provider are included in these conversations. They take place at the time of admission, during regularly scheduled care planning meetings, and when there is a change in the patient’s medical condition. A few DONs noted that the social workers in their facility are responsible for initiating the advance care planning conversations, and the nursing staff and physicians join in when additional clinical guidance is needed or to sign off on documentation.

Advance care planning conversations are often used to determine a resident’s preferences concerning hospitalization. DON 1 explained that during regular care planning meetings,

we could say to the family, we think we should really look at the palliative route right now ... and I think families are a little relieved when we say that we can do things here because they still want to be aggressive but not yet to the point that they are in ICU care and [there is] disruption from their normal routine.

In these cases, residents on palliative care receive symptom management care without a risky, troublesome hospitalization, while maintaining the option of hospitalization for other complications. DON 3 acknowledged that “everyone’s palliative is different.” Although some individuals diagnosed with a serious illness may want to continue with hospitalization or externally provided medical treatments, like chemotherapy or radiation, not every patient finds these measures beneficial. All of the DONs’ nursing homes offered residents the option to use external providers for palliative care and hospice services before the pandemic. The DONs explained that most of the palliative care consultations were delivered by a nurse practitioner specializing in palliative care or hospice, as ordered by the resident’s attending physician or recommended by the DON.

In some cases, a hospice provider coming into the nursing home to visit a resident will provide informal consultations for residents not enrolled in their services. DON 3 explained that “[hospice providers] bought into the team of the center so they would have a patient in bed A and they would also visit with bed B.” When an external provider “buys into” the nursing home’s nursing team, they form close relationships with the staff and may be willing to help staff treat other residents. They might consult with the nursing staff to give palliative care recommendations for complex cases when the patient does not qualify for or does not want hospice care. The DONs reported that these informal consultations often replaced referrals to palliative care specialists.

Informal consultations are powerful aids for managing complex pain and symptoms and for training staff on palliative care techniques. DON 1 explained that these consultations “are good education for my new nurses, as well [as for me], to always keep a handle on how to assess pain and adequately treat pain.” This is especially true when providers on staff would like to try alternative pain treatment modalities, such as massage and diathermy, in place of narcotics. It can be difficult to find the correct combination of these interventions, and the specialized external clinicians have more experience in this area and are able to guide staff on complex cases.

Most DONs expressed a desire for better education around pain management for all members of the care team, including physicians and nursing staff. They noted that even though pain should be seen as “the 5th vital sign” (Morone & Weiner, 2013), some nurses are not adequately trained to recognize pain and address it. Ideally, the nursing home medical director is fully trained to manage pain and can impart this knowledge to nursing staff, but some staff apparently did not take cues from the physicians and nurse practitioners. For example, DON 4 explained that

I had certain nurses that would [learn from physicians and nurse practitioners] and then would make their own suggestions, then we would talk about it. I had other nurses that would say to me, “well, they didn’t have any pain.” Well, how come she’s squirming then?

### Palliative Care Delivery During the Pandemic

A fundamental hurdle for palliative care at the start of the COVID-19 pandemic was the novelty of the disease and the resulting clinical uncertainty. DON 6 explained that “[COVID-19] seemed to affect more of the healthy ones. It did affect a couple of the palliative care patients ... but the portion of younger ones really kind of took me aback.” The DONs reported that COVID-19-positive residents often did not present with respiratory symptoms but rather migraines and gastrointestinal issues, such as diarrhea. It took time before staff was able to recognize these signs and shift their approaches to include increased surveillance on appetite and hydration.

Restrictions on external visits left nursing home staff with only internal resources to provide palliative care. Although Massachusetts regulations restricting nursing home visiting during the pandemic did not apply to medical practitioners (Massachusetts Office of the Governor, 2020), both the external providers and the nursing homes themselves discouraged or forbade these visits, out of fear of infection combined with some misunderstanding of the government restrictions. This meant that medical directors, nurse practitioners, and the nursing staff became fully responsible for caring for critically ill residents. These included residents near the end of life, whose care would normally be facilitated by external hospice providers. For example, DON 4 explained that

the nursing [staff] and I are doing their comfort care ... if I need guidance [from palliative care or hospice specialists], you do have to contract with them, but all of the contracts have gone to the wayside. So, the physicians and nurses come together for pain [management] or anything else we can suggest to help somebody and be there for them.

Despite losing this important resource, the DONs expressed satisfaction with the level of palliative care they were able to deliver given the circumstances. They found the nursing homes’ medical directors and nurse practitioners to be responsive to residents’ needs. Palliative care delivery during the COVID-19 pandemic was a collaborative effort between the nursing staff, the medical director, and any nurse practitioners. In the absence of guidance from palliative care specialists, the care team was often challenged to try out new approaches to see what might work best. DON 5, for example, said:

the nurse practitioner is pretty good about teaching and saying, “look out for this,” “look out for that.” The other thing we have found is that a lot of times [when someone] either keeps falling or keeps crawling out of bed [then], she’s a big proponent of going down and assessing them ... We’ve been [trying to not use narcotics] but some simple things that might make a difference, like a heating pad.

Some of the represented nursing homes had an in-house palliative care or hospice-certified medical director or nurse practitioner on staff during the crisis. These certified individuals were especially helpful in training DONs and the nursing staff to recognize and treat pain and symptoms of serious illness. This improved staff’s confidence in their ability to provide primary palliative care to residents. DON 2 explained that because her facility’s medical director was certified in palliative care, they could manage without visits from outside palliative care clinicians; the medical director could take over that role.

During the early stages of the pandemic, the lack of resources, including masks, gowns, pain medications, and other palliative care tools, was a significant barrier to palliative care delivery. Without these resources, the DONs felt that they had to reduce their interactions with residents because of the risk of exposure. This likely contributed to increased patient isolation and decline. In addition, some DONs reported that they had to adjust treatment plans to match the available resources, which they worried led to suboptimal care. For example, due to shortages of N95 masks, staff were not allowed to use nebulizers to relieve breathlessness for residents with asthma or other respiratory conditions, because of the risk to staff from airborne particles. This forced them to switch to inhalers, and because many clinicians made this switch, inhalers were in short supply across the region. In contrast, the two DONs who reported no problem with resources shared more positive feelings about their palliative care practice, despite having this responsibility thrust upon them during the COVID-19 pandemic.

### Eliciting Resident Goals of Care During the Pandemic

Determining patient goals of care through advance care planning is a critical ingredient of palliative care. The COVID-19 pandemic did not significantly alter the way staff approached advance care planning conversations, but it underscored the importance of understanding evolving patient goals of care during medical crises. The responsibility for these conversations remained the same as in pre-COVID times. Typically, the nursing staff and social services initiate conversations during admission and when there is a change in medical condition. The resident’s physician (often the medical director but sometimes represented by a nurse practitioner on staff) steps in when the nursing staff and social services are having trouble helping the family to understand the patient’s condition and the value of palliative care. Aware of the treatment restrictions necessitated by the pandemic, staff tried to elicit patient goals proactively. The staff reviewed every resident’s advance directive regardless of current COVID-19 status and discussed with residents and their families preferences for interventions like hospice, hospitalization, or intubation that might be ordered if they should become infected.

Advance care planning conversations were especially critical for residents who became COVID-19-positive. In some cases, a resident was initially asymptomatic but within days became seriously ill. The DONs found that COVID-19 affected each patient differently, which required staff to pay close attention to changes in the clinical situation and to update the residents’ families regularly. The nursing staff, social services, and the resident’s attending physician talked with families by phone or Zoom to explain the options for care. For example, DON 2 noted that care for many critically ill COVID-19-positive residents

was really just making sure people were comfortable. After all attempts to turn them around or them out of this situation, if they were not improving based upon what we did, then the conversation would shift to comfort, whether that means getting them something to ease their suffering or pain or just to calm them a little bit.

Because of the ban on family visits, staff became “the eyes and ears” for family members who had to make difficult decisions about their loved one’s care. Staff members would Zoom with residents’ families to give them a sense of the clinical situation so that they could make informed decisions regarding care. Although Zoom is by no means an adequate substitute for in-person family visits, video connections helped families have more effective advance care planning conversations and helped to keep them involved with care for their loved ones.

One area of advance care planning that saw significant change during the COVID-19 pandemic concerned the decision to hospitalize. A few of the interviewed DONs expressed apprehension about sending COVID-19-positive residents to the hospital for treatment. DON 1 explained that, in the early months of the crisis, the choice to avoid hospitalizing residents was

a choice made by the family members as well as the staff. To be honest, we would say that [the nursing home patients referred for hospitalization during the pandemic] are the lowest of the low on the totem pole to even get seen, and they would be on a stretcher for hours in the emergency room ... they are in their 90s, they have all these comorbidities, they are not going to make it.

In many cases, COVID-19-positive residents sent out to hospitals would “be made palliative” by the hospital clinicians who saw them there (DON 3) and would be sent back to the nursing home without being admitted to the hospital. In contrast, some COVID-positive residents admitted to the hospital were treated but were even less able to connect with their families there and risked dying alone, surrounded by unfamiliar staff. This risk to resident safety and well-being was very distressing to the DONs and spurred their efforts in the following months to keep residents in-house and treat them as best they could with the available knowledge and resources. DON 4 explained that they learned “so much going through it at that point, I made the determination at the facility level that we are not sending anybody else out—it’s do or die at the facility level.”

In addition, DONs noted an increase in the number of palliative care consultations that were done in the hospital when someone was admitted. They explained that before the COVID-19 pandemic in-hospital staff only addressed the residents’ chief concern, so that palliative care was unlikely to be prescribed or provided. In the current crisis, “most patients that have advanced comorbidities we’re finding now coming into the nursing home [from the hospital] all had palliative care consults ... so I’m assuming that’s an initiative they are engaged with because of COVID-19” (DON 2). Staff received information on the content of these consultations through medical records and were able to use them to direct care once the patient was back in the nursing home.

### Building Trust Between Residents, Family, and Nursing Home Staff

As reported by the DONs who participated in this study, the overwhelming crisis was accompanied by a deepening of the trust between nursing home staff and the residents and their families. Although it was always standard practice to include families in care planning when possible, during the pandemic the DONs felt that they had to put in extra effort to communicate with families about ongoing resident status because they knew that in-person visits would be strictly limited if a crisis arose. The DONs reported that they used Zoom technology and phone calls to give families regular updates, although they knew this could not compare to in-person visits. In describing these interactions, DON 3 said,

we are bringing [families] into the discussion. We’ve said, “this was a good week” or “this was a bad week” or we’ve started to say, “there’s been changes since you last saw them, and this is what we suggest” ... there’s been a couple that will say “no, not yet” but if we say it’s time to try this or that ... they will say “well you know her better.”

The DONs noted that families appreciated the work they were doing and trusted the nursing staff to provide good care, even while the nursing home was managing outbreaks. In many cases, families appreciated that the staff knew their loved ones better than clinicians in the hospital and chose to have their residents stay in the nursing home rather than being transferred out for care.

The formation of trust between residents, families, and staff mitigated suffering in three ways. First, open conversations about the realistic options for care helped to alleviate anxiety around the uncertain situation for residents and family members. Second, although staff could not replace support from family, trusted staff could stand in for family and provide comfort to residents isolated in the nursing homes. Finally, these relationships and open communication helped staff decide when to adhere strictly to infection control protocols and when to stray from them in order to balance the emotional and psychological well-being of residents and families.

### Impact of Resident Isolation on Palliative Care

Compassionate human interaction is a critical component of palliative and end-of-life care. The nursing home lockdowns and patient isolation, imposed to slow viral spread, led to significant decreases in well-being for residents in the nursing homes. Before the COVID-19 pandemic, residents would spend time in nursing home hallways, dining areas, and common spaces, providing some socialization with other residents. During the COVID-19 pandemic, residents were confined to their rooms to halt the spread of the virus. Residents became bored and suffered psychological and emotional distress after losing this socialization opportunity. DON 6 commented that this overall decline “is almost like a failure to thrive; it’s hard to have something to look forward to when you’re staring at the same four walls.” Another DON explained that she had noticed an increase in the use of antidepressants among her patient population, as well as an increase in the number of residents seeking palliative care, even among non-COVID-19 residents.

But most important was loss of interaction with family members. Even though the DONs reported that their facilities restricted family visits in the beginning of the COVID-19 pandemic, they allowed compassionate visits when a loved one was dying. DON 5 explained that

if someone is actively dying, we do allow families to come in. They do have to be screened and be escorted down. We have to educate [them on] the infection control practices, provide them with the appropriate equipment ... it depends on what stage in the dying process that somebody is. If it’s imminent, then we really allow them just as much time as they need.

A few of the DONs explained situations in which the families would “com[e] in through the back door” (DON 4). They could not justify allowing residents to die alone without the family present, despite the risk of infection these visits posed. They felt strongly that Zoom technology could not replace being with a loved one during their last moments, and they wanted to do everything in their power to support residents and families during the dying process.

## Discussion and Implications

The DONs’ reports prompt reflections on four aspects of nursing home palliative care delivery during the pandemic: the value of palliative care expertise and resources within the nursing home; the value of the external resources that were missing during the pandemic, while hospitals sometimes took on an unexpected role; the importance of flexible advance care planning allowing goals to evolve with changing circumstances; and the vital contribution to palliative care of human contact. These reflections suggest directions for research, policy, and practice.

### Internal Capacity for Palliative Care Delivery

Our findings align with the literature that suggests that palliative care challenges may be best overcome by increasing internal capacity for providing palliative care, as in-house staff are well situated to deliver this care (Carpenter & Ersek, 2021; Ersek et al., 2022). Through their everyday interactions with residents, staff learn about residents’ values and goals and could use this knowledge to inform patient-centered care. They are also able to monitor residents and could facilitate timely symptom management and support, given the needed time, technical knowledge, and access to drugs, devices, and supplies. Despite this potential, a lack of palliative care metrics, inadequate training, limited financial resources, payment structures, and quality measures favoring rehabilitation have curbed efforts to build in-house palliative care programs (Carlson et al., 2011; Carpenter et al., 2020). The DONs’ reports also underscore the importance of securing personal protective equipment and palliative care medications and resources early in a crisis to prevent delays in palliative care delivery. The impact of staff time and retention on internal capacity for integrated palliative care and development of trust between staff and residents and their families should be more thoroughly investigated. Public policy must commit to adequate resources for nursing home care if nursing homes are to supply appropriate palliative care.

### Access to External Palliative Care Expertise

Although in-house staff mustered palliative care resources during the pandemic, the DONs interviewed missed the advice of the hospice and palliative care consultants, underscoring their value. But palliative care consultations to nursing home residents were not widely available even pre-pandemic and are difficult to identify in claims data (Carpenter & Ersek, 2021; Miller, Lima et al., 2016). Future research considering access to palliative care should document how nursing homes reach out to external consultants post-COVID-19, with an eye to policy development to strengthen consulting relationships (Dunleavy et al., 2021). As other observers have noted, contact with hospice providers can support internal palliative care capacity by informing staff about pain and symptom management techniques (Meier, 2015). Additional research could describe the nature and prevalence of informal consultations with hospice providers and consider regularization of this pathway to palliative care capacity.

An incidental finding from our interviews, mentioned by a few DONs, concerns the role of the hospital in initiating palliative care plans for some nursing home residents during the pandemic. It is not standard practice for hospital staff to develop palliative care plans or recommendations as patients are discharged from hospital to nursing home, and when plans are present, they are not always followed. This may be due to the Medicare program’s expectation of rehabilitation within covered skilled nursing facility care or due to residents’ and families’ misunderstanding of the value of rehabilitation versus palliative care as patients transfer to or re-enter a nursing home (Carpenter et al., 2017, 2018; Honinx et al., 2020; Meier & Beresford, 2010). But during the pandemic, hospitals were often highly compromised in their ability to serve frail nursing home residents who were referred for admission. Palliative care assessments developed in the hospital emergency department diverted nursing home residents from hospital admission. They often identified return to the nursing home as the most realistic and preferred available course for these patients and recommended clinical palliative strategies. According to the DONs, in the absence of palliative care consultation in the nursing home, recommendations from hospital palliative care teams could inform posthospital resident care. With the growth of palliative care availability to all types of hospitalized patients, collaboration with hospitals might increase the access of nursing home residents to palliative care consultations (Cohen Marill, 2019; Reuter et al., 2019; Schoenherr et al., 2019; Southerland et al., 2020). More attention to palliative needs of residents in the transfer process could also reduce hospital readmissions and contain costs (Chang et al., 2021; McGarry & Grabowski, 2019; Zuckerman et al., 2016).

### Resident Goals of Care

Research into best practices for developing and adhering to residents’ goals of care through the extreme challenges of COVID-19 would provide evidence for quality metrics that capture the evolution of advance directives through changing prognoses and symptoms (Morrison et al., 2021). Clinicians with specialized palliative care expertise based within their nursing home (in contrast to unfamiliar external consultants) are well positioned to ensure that care continually aligns with evolving goals and wishes (Hickman et al., 2020). Educational programs could build on the pandemic experience that timely and honest conversations around options for care can better meet residents’ needs in a crisis and increase trust between families and staff.

### Social Contact: A Critical Element for Palliative Care

Observers have documented the devastating impact of nursing home lockdowns that isolated residents from fellow residents and from friends, family, and community visitors (neighbors, relatives, clergy; Bethell et al., 2021; Levere et al., 2021; Simard & Volicer, 2020). Not least of these impacts was the loss to palliative care of the social, psychological, and spiritual support of these relationships. Research and policy analysis have highlighted the immense value of family and other informal caregivers as a resource for community-dwelling older adults and persons with disability (Feinberg et al., 2021; Reinhard et al., 2019). Fewer studies have examined the substantial contribution of informal caregivers in residential care (Coe & Werner, 2022; Gaugler & Mitchell, 2022). Spurred by the “natural experiment” that removed this resource from the nursing home, future research should delineate the value provided by family members and other visitors in the provision of nursing home care, especially for residents experiencing distressing symptoms. This research could identify new ways to facilitate visits to nursing home residents with palliative care needs. Examples include counting visitors in requirements for scarce personal protective equipment, prioritizing family caregivers in vaccine distribution, and allowing residents to see family members and other visitors under controlled circumstances, even during lockdowns.

In a larger sense, a person-centered approach to resident life not only tailors direct care to resident preferences, but builds community within the nursing home and prioritizes residents’ connection to their larger community (family involvement, transportation to chosen religious services, visits from community groups, community outings and activities; Pioneer Network, 2022). Interaction with staff and other residents, and with the external community, was disrupted by the COVID-19 pandemic. A broad understanding of palliative care should include activities that support meaning and quality of life for nursing home residents.

### Strengths and Limitations

This study is exploratory but provides in-the-moment insight into the evolving, difficult situation nursing home staff faced during the COVID-19 pandemic. It aimed to gain a deeper understanding of some DONs’ experiences during this time and to enable their voices to be heard. As such, it adds to the literature on access to palliative care, stressing the dimension of effective resident centeredness, rather than resident outcomes.

The small sample size of self-selected DONs from one state and the reluctance of contacted DONs to participate during the pandemic are limitations of this study. The DONs who chose to be interviewed may have felt more comfortable talking about palliative care and may have had more time to participate compared with DONs who work in nursing homes with fewer resources, less focus on palliative care utilization, or more acute outbreaks of COVID-19. The nursing homes severely affected by COVID-19 had very high staff turnover, particularly for the DON position, which decreased their likelihood of inclusion in the study. Thus, the data may depict a more positive picture of how palliative care was delivered during the pandemic than was generally the case. Furthermore, DONs were asked about palliative care practices prior to the pandemic to capture their sense of how care had been affected. Recall bias may have affected their reports. This study, by seeking the views of DONs, also fails to capture the experiences of other staff delivering palliative care in nursing homes, or that of family caregivers, and residents, whose perspectives on palliative care may differ from the DONs.

Finally, this research was undertaken as an individual research project, so the same researcher (K.N.) both conducted the interviews and analyzed the data. Although intracoder reliability was improved by multiple rounds of coding and reexamination of data after theme development, researcher bias is possible given the absence of additional coders.

## Conclusion

Reports from nursing home nursing leadership in a time of pandemic provide a window on extreme challenges for nursing home palliative care. In excluding external consultants, family members, and other community visitors, pandemic restrictions brought the value of these external resources for palliative care into sharp focus. At the same time, the DONs we interviewed found and developed internal capacity to discern residents’ evolving goals of care and to manage the distressing symptoms of a devastating, highly contagious illness. Analysis of their views suggests that more can be learned from systematic examination of palliative care delivery during and after this crisis, especially by extending and deepening the person-centered lens that has been applied in some previous studies (Carpenter et al., 2020; Iida et al., 2021; Kaasalainen et al., 2019). Nevertheless, although there is always more to learn, existing evidence supports public policy to assure that nursing homes have both external and internal resources for palliative care and are accountable for providing it. The most important next steps for research, policy, and practice should be development and implementation of such initiatives and evaluation of their impact.

## Supplementary Material

igac030_suppl_Supplementary_Material
