# Supplementary material for: Nursing Home Palliative Care During the Pandemic: Directions for the Future
Source: Innov Aging. 2022 May 9;6(4):igac030. doi: 10.1093/geroni/igac030 (PMC9273407; doi:10.1093/geroni/igac030)
Supplement: igac030_suppl_Supplementary_Material [file igac030_suppl_supplementary_material.pdf]

# Online Supplemental Material

For: Nursing Home Palliative Care During the Pandemic: Directions for the Future

Kacy Ninteau, BS and Christine E. Bishop, PhD

https://doi.org/10.1093/geroni/igac030

**Interview Guide**

Thank you for agreeing to participate in this study. I'm hoping to learn about how patients in the facility you work at can access palliative care services. I will ask you about the nursing home you work in and your role there, the model of palliative care delivery used in this facility, how patient wishes for palliative care are documented and honored, and how the COVID-19 PHE has affected care delivery. My questions generally refer to long-stay nursing home patients, the people who live in your nursing home. If you would like to answer with respect to post-acute care patients, those admitted after a hospital stay for a shorter Medicare covered stay, please specify this in your answer. Do you have questions before we begin?

I. Participant and Facility Information
   - What is the name of the facility you work at?
   - What is your current position?

II. Physician Services
   - Can you give me a brief idea of how physician services are provided to patients in your facility?

III. Models of palliative care delivery before COVID-19 PHE
   - Can you tell me about how patients with severe pain and/or symptoms of serious illness were treated pre-COVID in your facility?
     - What were the roles of nursing staff, the patients' physician (or medical director), and any other specialty service?

    - Could you tell me about any special care that was ordered or provided for patients with severe symptoms of serious illness?
- Before the COVID-19 PHE, did patients at your facility have access to formal palliative services? By formal care, I mean specialty consults brought in from outside the nursing home, or personnel with specialty training who are employed by the nursing home.
- Is palliative care treated as different from hospice at your facility?
    - If yes, can you explain how you see the differences?
- Prior to COVID-19, was palliative care delivered by outside specialty providers, patients' physicians (or medical director), or in-house nursing staff working with a physician?
    - If utilized outside specialty providers, what was the referral process? What is the result of the typical consultation (symptom management process, ACP documentation, etc.)? Were these typically single consults or were there follow up visits?
    - If consults were with patients' physicians, what were the results of those visits (symptom management plans, ACP documentation, etc.)? Were they typically single consults or were there follow up visits?
    - If palliative care is delivered by in-house staff, who on staff is responsible? If relevant, do in-house nursing staff have special training beyond their professional training to deliver this service?
- Prior to the COVID-19 PHE, was it difficult for patients to receive palliative care services? Why/why not?
- Is there anything that has, or potentially could have, made delivering this type of care easier?
- Have some patients (or SNF patients?) who are admitted or readmitted to your facility from the hospital had an in-hospital palliative care consult? If yes, were you able to follow the care plan established by that consult? Why or why not?

IV. Use of advance care planning tools as they relate to palliative care

- Please describe briefly how advance care planning is carried out with patients in your facility. Who on staff is responsible for these conversations? What is the documentation process (proxy designation and completion of advance directives)?
- How are advance care planning conversations used to determine if palliative care will be appropriate for a resident? What is some information which may be given to a resident about palliative care during these conversations?
- In your opinion, could advance care planning and documentation be used to improve palliative care delivery?

V. Palliative Care during COVID-19 PHE

- How has your facility been affected by COVID-19?
- Did (does) your facility have enough resources to protect staff and patients from COVID-19?
    - If not, how did this affect patient care?

- How did facility lockdowns affect access to formal palliative care services? Were patients able to see palliative care providers and/or their other providers from outside the facility?
- During the COVID-19 PHE, were patients and their families engaged in ACP conversations with staff?
    - Did the content of these conversations change because of COVID-19?
    - How did staff address potential changes to access to palliative care during these conversations?
- Did any COVID-19 positive patients stay in your facility? Were they able to access palliative care services?
    - If yes, did these services differ in any way from palliative care for other serious illness (cancer, heart disease)?
- What if any guidance from regulatory agencies and/or the government was provided to you or your staff on how to provide palliative care during the COVID-19 PHE?
- Is there anything else you would like to share about delivering palliative care during the COVID-19 PHE that might help us and others understand what it was like in your facility during this time?

VI. Conclusion

- Before we end, is there anything else you would like to share with me?

## Method Details

### *Analytical Approach*

The thematic analysis approach was determined to be the best method for data analysis given the narrative nature of the interview data (Schoenberg & McAuley, 2007; Vaismoradi et al., 2016). The interview guide was informed by a literature review which highlighted key aspects palliative care delivery, including pain management, collaborations between outside providers and in-house staff, staff education, payment structures, resource availability during the COVID-19 pandemic, stigma around palliative care, etc. We hypothesized that palliative care delivery during COVID-19 was heavily influenced by pre-pandemic structures and processes in-place at the facility- and regulatory-levels. The thematic analysis approach allowed for both deductive and inductive coding and theme development (Vaismoradi et al., 2013). In some cases, codes and themes were directly related to our hypothesis. In other cases, codes and themes were derived from patterns which emerged unexpectedly in the data. The thematic analysis approach provided flexibility during the data analysis process, allowing for more data to be captured within our themes.

We will use our data around advance care planning to exemplify this deductive analysis. We knew from the literature review that advance care planning is a key aspect of high-quality palliative care. During the interviews, we asked targeted questions hoping to uncover how team dynamics and processes around advance care planning changed because of the COVID-19 pandemic. We present here the codes and themes derived from interview data following these questions:

### *Codes*

- Physicians stepped in to participate in advance care planning during COVID-19

- Physicians call families who may need additional support during COVID-19
- Staff updated advance directives with families to prepare for COVID-19

**Subtheme**: Staff communicate often with residents and families

**Theme**: Utility of Advance Care Planning During COVID-19 Pandemic

For the inductive analysis, we present our coding and theme development around trust formation as an example. The interview questions did not ask specifically about trust and relationship formation as they were more focused around processes and outcomes related to advance care planning. Yet, the narratives highlighted the role of trust between residents, families, and staff in coordinating care for all residents during the pandemic, not just care for residents with COVID-19. We present here the codes which induced the development of the theme, "Trust Formation Between Residents, Family, and in-house Staff During COVID-19,"

- Quality relationship between residents and staff improves palliative care
- In-house palliative care better than hospice because staff know resident
- Staff act as primary caregiver during COVID-19 lockdowns
- Staff are "eyes and ear" for family during lockdown
- Families trust staff will know what care is best for resident during lockdown

### *Data Analysis Procedure*

During the initiation phase, the interviews with transcribed verbatim with all potentially identifying information removed, such as the name of the participant and nursing home. K.N. read through the data multiple times to get a sense of the whole and the key issues at play. Reflective notes were taken after immersion in the data which captured K.N. initial understanding of the data. These notes served to provide a space for researcher reflection, reflexivity, and questioning. Trends which emerged from these notes were traced back to key quotes in the transcripts.

Once key quotes were identified, they were reduced to codes to be more manageable. The codes used could be classified into two main categories: conceptual codes, which identified key elements of the palliative care delivery process, and participant perspective codes, which related to the DONs' perception of their experiences and their role within critical interpersonal relationships.

During the construction phase, the codes were evaluated and compared. Codes around the different aspects of palliative care delivery were sorted together. Codes which related to palliative care before the COVID-19 pandemic and care during the pandemic were separated to be able to compare the two situations. Codes were reevaluated multiple times and compared to the corresponding quotes in the transcripts to ensure that they truly captured the data. K.N. then made judgements on the frequency, importance, and content of each code to abstract subthemes and themes. These subthemes and themes were evaluated to see how they related to the overall research question. It was determined that the subthemes and themes abstracted during the analysis were helpful in conceptualizing how palliative care had changed during the COVID-19 pandemic and how the DONs had reacted to these changes.

Next, K.N. stepped away from the data, subthemes, and themes for some time to create distance. When returning to the data, K.N. conducted a reexamination to see the data with a new perspective and self-correct any incongruences. There were a few cases were the original subthemes and themes did not seem to fully capture the data. In these cases, subthemes and themes were redefined to be clearer or were split into more than one theme or subtheme to be more precise. Finally, the established themes and subthemes were compared to established

knowledge and literature, which allowed for the results to be situated within the broader context of nursing home care and structure.

Through these processes of rectification and finalization, a story line was developed which captures the DONs' narratives around the changes to palliative care delivery and how their work and relationships evolved during this time.

In the table below, we provide some illustrative codes used for subtheme and theme development.

| | | |
|---|---|---|
| Pre-Pandemic Palliative Care Practices | How DONs Define Palliative Care | • comfort care<br>• EOL run by in-house staff<br>• Hospice is stigmatized, palliative care is nicer wording<br>• Care oriented to resident's goals and wishes<br>• Hospitalizations disrupt routines, in-house palliative care can still be aggressive |
| | Pain Management | • Hospice providers may overuse opioids<br>• Pain usually not well managed on narcotics<br>• Alternative interventions exist but are not used often<br>• DONs see benefit in using alternative interventions, not trained to use them |
| | Role of Outside Palliative Care and Hospice Providers | • Outside providers "buy into" in-house team<br>• Outside providers informally consult for residents not on their service<br>• Outside providers assist DONs in trying alternative pain management methods<br>• Nursing homes contract with outside palliative care and hospice providers |
| | Staff Education | • Nursing staff not trained enough to recognize pain<br>• Staff not trained on nuances of advance care planning<br>• Outside providers teach in-house staff novel pain management approaches<br>• DONs wish they had more palliative care training in school |
| Palliative Care Delivery During COVID-19 Pandemic | Role Designation with Loss of Access to Outside Palliative Care | • Informal consults with outside providers stopped during COVID-19<br>• DONs and nurse practitioners collaborate to try new pain management interventions<br>• Extra support available when medical director is hospice and palliative care certified |
| | Care for Residents with COVID-19 | • COVID-19 affected younger, healthier as much as palliative care patients<br>• COVID-19 presented as symptoms like diarrhea and migraines, rather than respiratory issues<br>• Focus on making residents comfortable<br>• Limited COVID-19 treatments early in pandemic |

| | | |
|---|---|---|
| | Resource Availability | • Shortages of medication and PPE at the start of pandemic<br>• Treating residents with COVID-19 became easier with more resources<br>• No nebulizer use without N95 masks for staff<br>• DONs responsible for buying supplies<br>• Large hospital systems buy-up most supplies |
| Eliciting Resident Goals of Care During the Pandemic | Staff Communication with Residents and Families | • Team-based advance care planning<br>• Social services involved in advance care planning<br>• "Drive-by," in-parking lot advance directive completion during facility lockdowns<br>• Staff communicate with families about treatments available in-house<br>• Physicians stepped in to participate in advance care planning during COVID-19<br>• Physicians call families who may need additional support during COVID-19<br>• Staff updated advance directives with families to prepare for COVID-19 |
| | Care planning for COVID-19-Positive Resident | • Status of COVID-19-positive residents change quickly<br>• Care planning done in advance to ensure family was prepared<br>• DONs suggested palliative care route to large proportion of COVID-19-positive residents<br>• Rapid transition of COVID-19-positive patients from asymptomatic to symptomatic |
| | Hospitalization | • Few hospitalizations because nursing home residents were not a priority<br>• DONs worried that hospitalized patients would die alone<br>• Hospitalized residents "made palliative" and sent back |
| Building Trust Between Residents, Families, and Nursing Home Staff | | • Quality relationship between residents and staff improves palliative care<br>• In-house palliative care better than hospice because staff know resident<br>• Staff act as primary caregiver during COVID-19 lockdowns<br>• Staff are "eyes and ear" for family during lockdown<br>• Families trust staff will know what care is best for resident during lockdown |
| Impact of Resident Isolation on Palliative Care | | • Increased use of anti-depressants<br>• Failure to thrive<br>• Declines in health and increased hospice use even in COVID-19-negative residents<br>• DONs wonder about long-term impact of isolation |

### *Justification for single coder*

The literature reports that it is standard practice to have 2-3 researchers conduct interviews and to code the data to mitigate researcher bias. Intercoder reliability is a golden standard of qualitative research (Vaismoradi et al., 2016). Given that this study was undertaken as a thesis project, we were unable to include a second coder during the data analysis process. K.N. conducted multiple rounds of coding to improve intracoder reliability. K.N. also went back and reexamined the data after spending significant time on theme development. This served as a check that the themes aligned with the data and that key aspects had not been missed during the analysis. Despite these measures, the use of a single coder remains a limitation of the study (Tracy, 2010).

## References Cited


Schoenberg, N. E., & McAuley, W. J. (2007). Promoting Qualitative Research. *The Gerontologist*, *47*(5), 576-577. https://doi.org/10.1093/geront/47.5.576

Tracy, S. J. (2010). Qualitative Quality: Eight “Big-Tent” Criteria for Excellent Qualitative Research. *Qualitative Inquiry*, *16*(10), 837-851. https://doi.org/10.1177/1077800410383121

Vaismoradi, M., Jones, J., Turunen, H., & Snelgrove, S. (2016). Theme development in qualitative content analysis and thematic analysis. *Journal of Nursing Education and Practice*, *6*, 100-110. https://doi.org/10.5430/jnep.v6n5p100

Vaismoradi, M., Turunen, H., & Bondas, T. (2013). Content analysis and thematic analysis: Implications for conducting a qualitative descriptive study. *Nursing & Health Sciences*, *15*(3), 398-405. https://doi.org/https://doi.org/10.1111/nhs.12048
